# The Influence of Social Media on Melanoma Prevention: A Retrospective Analysis

**DOI:** 10.7759/cureus.92408

**Published:** 2025-09-15

**Authors:** Entela Shkodrani, Alert Xhaja, Sabina Dedej, Barbara Shkodrani, Gloria Hoxhallari

**Affiliations:** 1 Department of Dermatology, University Medical Center of Tirana "Mother Teresa", Tirana, ALB; 2 Department of Biomedicine and Prevention, University of Rome Tor Vergata, Rome, ITA

**Keywords:** dermoscopy, impact, melanoma, skin cancer awareness, social media

## Abstract

Background

The widespread use of social media has introduced novel opportunities for public health communication, particularly among adolescents and young adults. In dermatology, these platforms are increasingly employed to disseminate information on skin cancer prevention and early detection. Given the rising incidence of melanoma in younger populations, social media represents a strategic tool to enhance awareness, promote protective behaviors, and support early screening initiatives.

Aim

This study aims to explore the main referral sources for dermoscopic examination in patients with multiple melanocytic nevi (MMN) and to assess the role of social media across different demographic groups.

Methods

A retrospective study was conducted on 144 patients with >10 melanocytic nevi undergoing dermoscopy for the first time at a private dermatology clinic in Tirana, Albania, between 2023 and 2024, selected from a total of 1,103 screened patients. Dermoscopic examinations were performed using the FotoFinder Vexia Medicam 1000s, and images were evaluated by an experienced dermatologist based on international diagnostic criteria. Statistical analyses included Student’s t-test, Chi-square test, and one-way ANOVA; p < 0.05 was considered statistically significant.

Results

Among 1,103 patients, 144 met the inclusion criteria. The cohort had a mean age of 30.49 ± 10.34 years and was 68% female. The leading referral source was social media exposure (31.7%), followed by dermatologist referrals (22.5%) and personal concern about mole changes (16.9%). Older patients were more often referred by dermatologists (35.56 ± 12.52 years; p = 0.001) or presented due to personal concerns (34.50 ± 9.93 years; p = 0.036), whereas younger patients more frequently sought dermoscopy due to social media exposure (26.53 ± 8.01 years; p = 0.001). Men were more likely to be referred by dermatologists (37% vs. 16.5%; p = 0.010), while women were more likely to seek dermoscopy based on self-observed mole changes (21.6% vs. 6.5%; p = 0.030).

Conclusion

Social media plays a significant role in motivating younger individuals to seek dermoscopic evaluation, while older adults are more influenced by clinical referrals or personal concern. The observed age and gender differences in referral pathways underscore the importance of targeted digital health strategies to promote early melanoma detection. However, as this study was a single-center retrospective analysis with a limited sample size, the findings should be interpreted cautiously. Larger, prospective studies are needed to confirm these results and assess their broader public health implications.

## Introduction

The global growth of social media and the Internet in the past two decades has significantly impacted the dissemination of public health information. Public health initiatives have increasingly leveraged social media platforms to raise awareness and promote the prevention of various diseases by improving access to health information in several clinical specialties [1]. With regard to dermatology, social media has been employed by both healthcare professionals and patients, fostering a bidirectional exchange of information and engagement [2]. Platforms such as Facebook [3], YouTube [4], Twitter [5], and Instagram [6] are utilized for a variety of purposes, including health education, marketing, crowdsourcing, community building, and screening initiatives. Despite ongoing prevention efforts, rates of melanoma and non-melanoma skin cancers continue to rise in the United States and globally [7], particularly among adolescents and young women.

Melanoma - the deadliest form - is the second most common cancer in individuals aged 15-29, accounting for 8% of diagnoses in those aged 15-19 and 18% among those aged 25-29 [8]. Melanoma is a malignant tumor arising from melanocytes, the pigment-producing cells of the skin. It is characterized by uncontrolled melanocytic proliferation, often triggered by DNA damage from ultraviolet (UV) radiation [9]. Melanoma diagnosis relies primarily on clinical evaluation, with diagnostic accuracy increasing from approximately 70% with visual inspection alone to up to 90% when supported by dermoscopy. However, histopathological examination remains the gold standard for the definitive diagnosis of melanoma [10]. Early detection is vital, as stage I melanoma has a five-year survival rate of 98%, while metastatic melanoma shows a significantly lower rate of 35%; however, outcomes are improving with the advent of immunotherapy [11].

Given that young people spend over eight hours a day on social media, these platforms represent a powerful channel for disseminating information and promoting melanoma prevention strategies. In fact, social media plays a pivotal role in promoting sun protection and raising awareness about the risks of indoor tanning, particularly among adolescents and young adults [12]. The aim of this study is to explore the main referral sources for dermoscopic examination in patients with multiple melanocytic nevi (MMN) and to assess the role of social media across different demographic groups.

This study was presented as an oral presentation at the Annual Dermatology Conference in Skopje.

## Materials and methods

This study was conducted at a private dermatology clinic in Tirana using retrospective data obtained from patient registers covering the period January 2023 to December 2024. The clinic does not operate under an institutional review board (IRB); therefore, no formal IRB approval was sought. All data were anonymized prior to analysis, and no identifiable patient information was used. Written informed consent for dermoscopic examination and use of clinical data for research purposes was obtained from all adult participants; for minors, consent was provided by a parent or legal guardian. Inclusion criteria consisted of patients presenting with more than 10 melanocytic nevi (MMN) and undergoing dermoscopy for the first time. Exclusion criteria include the following: (1) history of previous dermoscopic or histopathological evaluation for melanoma, (2) known diagnosis of melanoma or other skin cancers, (3) incomplete medical records, and (4) refusal to provide consent. Demographic (age and sex), anthropometric (height, weight, and BMI), and clinical data were collected during standardized clinical evaluation at the time of dermoscopy. Because the primary focus of this study was referral pathways, anthropometric data were not analyzed further but are available upon request.

Referral source was assessed through a standardized patient intake question: “What prompted you to seek a dermoscopic examination?” Respondents selected from a predefined list of options, including social media exposure, dermatologist referral, personal concern, general physician referral, routine check-up, television, and other sources. Dermoscopic examinations were performed using the FotoFinder Vexia Medicam 1000s device. Standardized dermoscopic terminology was applied according to international consensus guidelines [13]. Both macroscopic and dermoscopic digital images of suspicious lesions were obtained and stored securely under anonymized codes. All images were evaluated by a board-certified dermatologist with more than 20 years of experience using established diagnostic criteria [14]. For subgroup analyses, patients were stratified into younger (≤30 years) and older (>30 years) categories. The cutoff was based on the median age of the study cohort. Statistical analyses were performed using SPSS software (IBM Corp., Armonk, NY). Continuous variables were expressed as mean ± standard deviation (SD), while categorical variables were summarized as counts and percentages. Student’s t-test was used to compare the mean age between groups. Chi-square tests were applied to analyze associations between gender and referral pathways. One-way ANOVA with Bonferroni post hoc testing was performed to assess age differences across all referral pathways. Results are presented with 95% confidence intervals (CI); CIs not crossing zero were considered statistically significant for mean differences. A p-value < 0.05 was considered significant.

## Results

Between 2023 and 2024, a total of 1,103 patients underwent dermoscopic examination at our clinic. Of these, 144 patients met the inclusion criteria for MMN and were included in the study. The patients' ages ranged from 6 to 63 years, with a mean age of 30.49 ± 10.34 years (Table 1). The cohort consisted of 97 females (67.8%) and 47 males (32.2%), as shown in Table 2.

**Table 1:** Age distribution overview

	N	Minimum value	Maximum value	Average value	Standard deviation
Age	144	6	63	30.49	10.336

**Table 2:** Demographic profile: gender

Gender	Frequency (N)	Percentage (%)
Female	97	67.8
Male	47	32.2
Total	144	100.0

**Figure 1:**
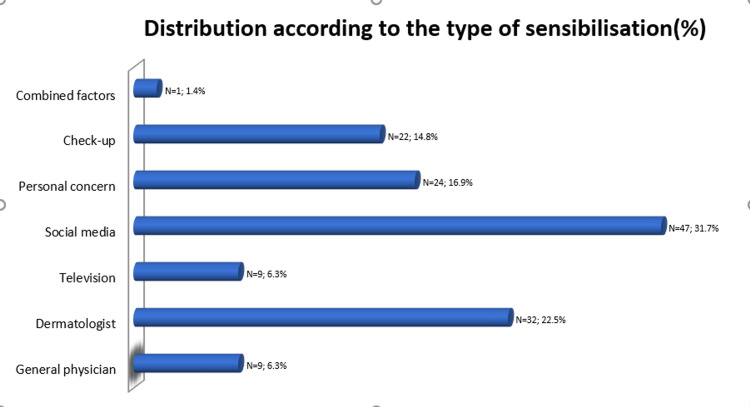
Distribution of referral sources for dermoscopic examination by percentage and number of patients

The most frequently reported reason for undergoing dermoscopy was social media (N = 47; 31.7%). This was followed by referrals after appointments with a dermatologist (N = 32; 22.5%), personal concern about changes in a mole’s appearance (N = 24; 16.9%), regular check-ups (N = 22; 14.8%), and exposure to information via television programs (N = 9; 6.3%). A small proportion of cases was influenced by general physicians (N = 9; 6.3%) or other combined factors (1.4%), as shown in Figure 1.

Question 1: Are there differences in the average age of patients based on the type of sensitization?

The data analysis used the Student’s t-test to explore the relationship between the average age of patients and the different referral pathways for dermoscopy (Table 3). Older individuals were more likely to undergo dermoscopy after being referred by a dermatologist (mean age: 35.56 ± 12.518 years, p = 0.001) or due to personal concern (mean age: 34.50 ± 9.926 years, p = 0.036). Younger individuals (mean age: 26.53 ± 8.005 years) showed a higher tendency to perform dermoscopy after exposure to social media posts (p = 0.001). No significant age differences were found for patients referred by general physicians (p = 0.457), television (p = 0.958), or routine check-ups (p = 0.134).

**Table 3:** Representation of the number and mean age of individuals with different types of sensitizations (Student’s t-test) Data are presented as the number of patients (N), mean age ± standard deviation (SD), p-values (significant at p < 0.05), t-values, and 95% confidence intervals (CIs) for each referral source leading to dermoscopic examination.

Referral	Number of patients (N)	Age (years) Mean ± SD	p-value	95% CI lower limit	95% CI upper limit	t-value
General physician	9	28.00 ± 8.874	0.457	-4.39	9.71	0.746
Dermatologist	32	35.56 ± 12.518	0.001	-10.53	-2.58	-3.265
Television	9	30.67 ± 5.148	0.958	-7.25	6.87	-0.053
Social media	47	26.53 ± 8.005	0.001	2.41	9.46	3.329
Personal concern	24	34.50 ± 9.926	0.036	-9.35	-0.31	-2.113
Check-up	22	27.45 ± 9.811	0.134	-1.12	8.31	1.506

Question 2: Are there gender differences in the type of sensitization to perform dermoscopy?

The Chi-square test was used to examine gender differences across the various referral pathways (χ² = 7.36; p = 0.010), as shown in Table 4. Men were significantly more likely to perform dermoscopy when referred by a dermatologist (37% vs 16.5%; p = 0.010). Women, on the other hand, were more likely to undergo dermoscopy due to personal concern about changes in a mole’s appearance (21.6% vs 6.5%; p = 0.030). No significant gender differences were observed for referrals from social media, general physicians, television, or check-ups.

**Table 4:** Distribution of referral sources for dermoscopic examination by gender, presented as absolute numbers (N) and percentages. Chi-square (χ²) test statistics and p-values (p < 0.05) are reported to evaluate gender-related differences across referral categories.

		Gender		Total	χ^2^	p-value
		Female	Male			
General physician	N	7	2	9	0.435	0.719
	%	7.2%	4.3%	6.3%		
Dermatologist	N	16	17	33	7.359	0.010
	%	16.5%	37%	23.1%		
Television	N	5	4	9	0.663	0.469
	%	5.2%	8.7%	6.3%		
Social media	N	32	15	47	0.002	1
	%	33%	32.6%	32.9%		
Personal concern	N	21	3	24	5.113-	0.030
	%	21.6%	6.5%	16.8%		
Check-up	N	17	5	22	1.062	0.457
	%	17.5%	10.9%	15.4%		

Question 3: Is there a difference in average age across the various sensitization pathways?

A one-way ANOVA with post hoc Bonferroni testing was performed to assess whether the mean age of patients differed according to the referral pathway for dermoscopic examination. The analysis demonstrated a statistically significant difference among groups (F(6,137) = 4.279; p = 0.001), as shown in Table 5.

**Table 5:** Multiple comparison of mean age across referral pathways (Bonferroni test) NS: Not significant; *p < 0.05 was considered significant.

Comparison	Mean difference (years)	Std. error	p-value	95% CI (lower to upper)
Dermatologist vs social media	9.47	2.26	0.001*	2.48 to 16.47
Dermatologist vs check-up	8.78	2.73	0.035*	0.31 to 17.25
Social media vs personal concern	–7.81	2.45	0.037*	–15.39 to –0.23
Other comparisons	NS		>0.05	

Patients referred by a dermatologist were significantly older than those influenced by social media (p = 0.001) and those attending for a routine check-up (p = 0.035). Patients influenced by social media were significantly younger compared to those presenting due to personal concern (p = 0.037). No other pairwise comparisons reached statistical significance (p > 0.05).

For additional clarity, patients were stratified into two groups: younger (≤30 years) and older (>30 years). Younger patients were more frequently influenced by social media exposure, while older patients were more commonly referred by a dermatologist. These differences were statistically significant (Chi-square test, p < 0.05).

Question 4: Are there gender differences across referral pathways?

**Figure 2:**
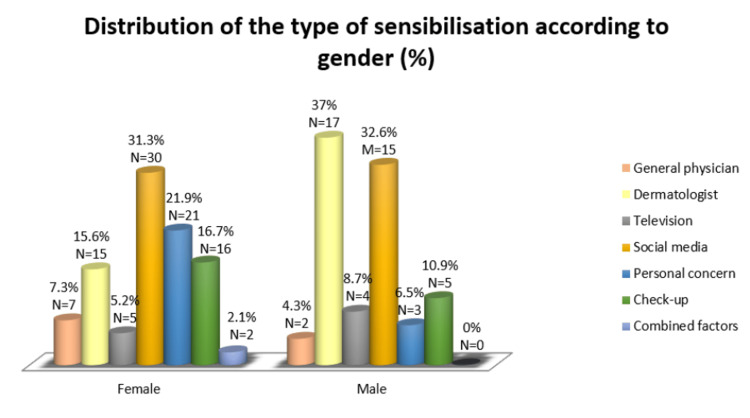
Gender-based differences in referral sources for dermoscopic examination

A Chi-square test confirmed statistically significant gender differences (χ² = 13.32; p = 0.038) in the distribution of referral pathways. Men were more likely to perform dermoscopy based on referrals from a dermatologist (37%, N = 17). Women were more inclined to perform dermoscopy after noticing changes in their mole’s appearance (21.9%, N = 21), as shown in Figure 2.

## Discussion

Social media has become a transformative force in modern medicine, providing a dynamic platform for the dissemination of health-related information to the public on a wide range of medical concerns and diseases. It also serves as a powerful tool for healthcare professionals to raise awareness, support health education, and engage with broader audiences. These platforms offer diverse features - including posts, stories, reels, polls, and question boxes - that enhance communication and outreach. Recent studies have demonstrated a dramatic shift in how patients engage with medical information, with over 80% of dermatology patients reporting the use of the Internet as a health resource - primarily prior to consulting a physician [15]. Platforms such as Google, YouTube, and Facebook were most frequently accessed, and female patients with higher education levels and greater disease-related anxiety were more likely to search online.

Melanoma and non-melanoma skin cancers constitute a significant portion of global cancer diagnoses, with incidence rates steadily increasing - particularly among fair-skinned populations with prolonged UV exposure [16]. Survival outcomes in malignant melanoma are strongly linked to early detection; in situ melanoma poses no risk of mortality, and thin lesions carry minimal metastatic potential [17]. Given this, skin cancer prevention initiatives are crucial, and social media plays an increasingly vital role in raising awareness, educating the public, and promoting early detection through targeted and widely accessible campaigns. About 90% of non-melanoma and 85% of melanoma skin cancers are caused by UV radiation from the sun and indoor tanning, yet many remain unaware of this risk [18]. A CDC survey found that over half of adults experienced sunburn in the past year, indicating poor sun protection [19]. Indoor tanning is especially popular among young people, contributing to thousands of new skin cancer cases annually. Social media networks are proven to be a powerful tool for spreading awareness about skin cancer risks and primary prevention, particularly related to ultraviolet and tanning bed use [20,21]. Our study explores the role of social media in secondary prevention, specifically in encouraging individuals to seek dermoscopic evaluation.

In our study of 144 cases, we found that 31.7% of patients underwent dermoscopy after becoming aware of skin cancer risks through social media, with the majority being younger individuals. Older patients tend to rely more on professionals (e.g., dermatologists) or personal concerns, indicating that their decision-making may be driven by clinical advice or observable changes in their skin. Conversely, younger individuals are more likely to seek dermoscopy after exposure to online content, reflecting a trend toward digital influence in health-seeking behaviors. Social media is an increasingly important site for younger individuals’ acquisition of health information [22]. The European questionnaire on Information and Communication Technologies Data [23] reveals that there exists a disparity between the Internet usage of people of different age groups. About 61% of Internet users in the United Kingdom between 16 and 24 years responded that they used the Internet to search for health information online, 18% more than five years earlier. In 2020, 70% of the 24- to 25-year-olds also informed themselves about health issues online. People aged 55-64 were least likely to research health information online, with 57% responding that they used the Internet for this purpose.

Gender differences also emerged in this study, with men more frequently relying on dermatologist referrals, whereas women tended to act based on personal observation of mole changes. Men have higher levels of skin cancer incidence and mortality than women [24,25]. This disparity is mainly due to their poorer use of primary and secondary prevention strategies. Studies show that men engage in self-examination less frequently and are more likely to delay reporting cancer symptoms to a doctor. These findings suggest the need for tailored public health strategies to increase skin cancer awareness across different demographics.

While specific platform data were not systematically collected, informal clinical observations suggest that Instagram and Threads were frequently cited by younger patients, while Facebook and YouTube appeared more common among older individuals. Future studies should incorporate structured assessments of platform type and engagement frequency to better characterize the digital referral landscape.

Limitations of the study

This study has several limitations that should be acknowledged. First, it was conducted at a single private dermatology clinic, which may limit the generalizability of the findings to other populations or healthcare settings. Second, the analysis was restricted to age and gender, while other potentially relevant factors, such as socioeconomic status, educational level, digital access, and health-seeking behaviors, were not assessed and may act as important confounders. Third, the sample included only patients with MMN undergoing dermoscopy for the first time, which introduces a selection bias and may not reflect the broader population at risk. The sample also exhibited an unbalanced gender distribution, with a predominance of female participants, which may have affected gender-related comparisons. Finally, although social media emerged as an important referral pathway, the type of platform, frequency of exposure, and credibility of the source were not evaluated, limiting the depth of conclusions about its specific impact.

Despite these limitations, the study provides valuable exploratory insights into the role of social media in influencing health-seeking behaviors for melanoma prevention in Albania and highlights the need for larger, multicenter studies that incorporate additional demographic, social, and behavioral variables.

## Conclusions

This study highlights the growing impact of social media as a referral source for dermoscopic examination, particularly among younger individuals with MMN. The findings reveal clear age- and gender-related differences in health-seeking behavior, with younger patients more influenced by digital content and older patients relying more on clinical referrals or personal concerns. While social media emerged as the leading trigger for dermoscopy in this cohort, these results should be interpreted cautiously due to the limited sample size, single-center design, and absence of longitudinal follow-up. Nevertheless, these preliminary insights suggest that targeted, demographically tailored awareness campaigns on social media platforms hold potential as part of secondary prevention strategies for melanoma. Future large-scale, multicenter, and prospective studies are warranted to evaluate the effectiveness, sustainability, and broader public health impact of such interventions.
